# Pleural Toxocariasis Presenting as Eosinophilic Pleural Effusion: A Case Report

**DOI:** 10.7759/cureus.109990

**Published:** 2026-05-31

**Authors:** Mohamed Lemine Sidi Oumar, Sidi Mohamed Limame, Mariem Vall Maleck, Hachem Taleb Elemine, Mohamed Lemine Cheikh Brahim Ahmed

**Affiliations:** 1 Department of Pulmonology, National Hospital Center, Nouakchott, MRT; 2 Department of Cardiology, Centre National de Cardiologie, Nouakchott, MRT; 3 Department of Thoracic Surgery, National Hospital Center, Nouakchott, MRT; 4 Research Unit, National Hospital Center, Nouakchott, MRT

**Keywords:** albendazole, eosinophilic pleural effusion, pleural eosinophilia, pleural toxocariasis, toxocara canis

## Abstract

Eosinophilic pleural effusion (EPE) is a rare pleural disorder characterized by a significant proportion of eosinophils in the pleural fluid. Its etiologies are diverse and include malignancy, infections, pulmonary embolism, drug-induced reactions, and parasitic diseases. Pleural involvement in toxocariasis is rare and may pose diagnostic challenges, particularly in tuberculosis-endemic regions.

We report the case of a 67-year-old male farmer from a rural area who presented with progressive dyspnea, right-sided chest pain, fever, and general deterioration. Physical examination revealed signs consistent with a right pleural effusion. Laboratory investigations showed leukocytosis with peripheral eosinophilia (1,040/μL; reference range: 0-500 cells/μL) and elevated inflammatory markers. Chest imaging demonstrated a loculated right pleural effusion.

Pleural fluid analysis revealed an exudative effusion with marked eosinophilia. Microbiological investigations, including bacterial cultures and acid-fast bacilli testing, were negative, although pleural tuberculosis could not be completely excluded based on microbiology alone. Cytological examination showed no malignant cells; however, a single negative cytology does not fully exclude malignancy. Despite pleural drainage and empirical broad-spectrum antibiotic therapy, no sustained clinical improvement was observed.

Further investigations revealed elevated anti-Toxocara canis IgG levels in both serum and pleural fluid, confirmed by Western blot analysis. Given the clinical context of EPE, negative microbiological workup, and persistent eosinophilia, these findings supported a diagnosis of pleural toxocariasis, although seropositivity may also reflect prior exposure. Treatment with albendazole was followed by clinical and radiological improvement, with resolution of eosinophilia and marked regression of the pleural effusion.

This case highlights the importance of including parasitic infections, particularly toxocariasis, in the differential diagnosis of EPE. In tuberculosis-endemic regions, careful evaluation is required to avoid premature diagnostic closure. Albendazole therapy may be associated with clinical improvement in selected cases; however, causality cannot be definitively established from a single case report.

## Introduction

Eosinophilic pleural effusion (EPE), defined by the presence of ≥10% eosinophils in pleural fluid, is an uncommon clinical entity, accounting for approximately 5-16% of exudative pleural effusions [1,2]. Its etiologies are heterogeneous and include air or blood in the pleural space, malignancies, pulmonary embolism, infections, drug-induced reactions, and parasitic diseases [3,4]. Importantly, malignancy remains one of the leading causes of EPE, particularly in older patients, and must be systematically excluded in the diagnostic workup [1,3].

Parasitic infections represent a well-recognized but frequently underdiagnosed cause of EPE, particularly in endemic and rural settings [4,5]. Among these, toxocariasis is a zoonotic disease caused by Toxocara canis or Toxocara cati, commonly associated with visceral larva migrans and peripheral eosinophilia [5,6]. Pulmonary involvement has been increasingly reported with the wider use of thoracic imaging and serological testing; however, pleural manifestations remain rare, with only a few cases reported in the literature [6,7].

The diagnosis of pleural toxocariasis is particularly challenging due to its nonspecific clinical, biological, and radiological features. It may mimic more common conditions such as parapneumonic effusion, empyema, malignancy, or pleural tuberculosis, especially in tuberculosis-endemic regions [8,9]. Importantly, pulmonary embolism and other common causes of EPE must also be considered and excluded before rare etiologies are retained. In such settings, initial microbiological investigations are often negative, and empirical antibiotic therapy may delay the diagnosis.

Pleural toxocariasis should therefore be considered in patients with EPE when common causes are excluded and there is poor response to standard antimicrobial therapy.

## Case presentation

A 67-year-old male farmer living in a rural area presented with progressive dyspnea, right-sided chest pain, fever, asthenia, and anorexia. His medical history was notable for poorly controlled hypertension treated with amlodipine.

On admission, the patient was febrile and tachypneic. Physical examination revealed signs consistent with a right pleural effusion, while the remainder of the examination was unremarkable.

Laboratory investigations demonstrated leukocytosis with peripheral eosinophilia, elevated inflammatory markers, and severe microcytic anemia suggestive of iron deficiency (Table 1). Liver and renal function tests were within normal limits. Procalcitonin was elevated (2 µg/L), although subsequent microbiological investigations, including blood cultures, remained negative.

**Table 1:** Laboratory findings Laboratory investigations showed leukocytosis with peripheral eosinophilia, severe microcytic anemia, and elevated inflammatory markers, while liver and renal function tests remained within normal limits.

Blood Test	Value	Reference Range (Unit)
Alanine aminotransferase (ALT)	31	7–56 U/L
Aspartate aminotransferase (AST)	21	10–40 U/L
Alkaline phosphatase	67	44–147 U/L
Albumin	4.6	3.5–5.5 g/dL
Total bilirubin	0.7	0.1–1.2 mg/dL
Gamma-glutamyl transferase (GGT)	19	9–48 U/L
Total protein	6.2	6.0–8.3 g/dL
Prothrombin time	11	10.9–12.5 seconds
Estimated glomerular filtration rate (eGFR)	113	>90 mL/min/1.73 m²
Blood glucose	4.8	3.9–5.5 mmol/L
Calcium	8.2	8.5–10.5 mg/dL
Vitamin D	45	30–50 ng/mL
Hemoglobin	7.2	13.8–17.2 g/dL
Mean corpuscular volume (MCV)	59	80–100 fL
White blood cell count	14,180	4,000–11,000/µL
Absolute eosinophil count	1,040	0–500 cells/µL
Platelet count	400,000	150,000–450,000/µL
C-reactive protein (CRP)	18	<5 mg/L
Sodium	144	135–145 mEq/L
Potassium	4.0	3.5–5.0 mEq/L
Chloride	103	96–106 mEq/L
Procalcitonin	2	<0.05 µg/L
Blood cultures	Negative	Negative

Chest radiography demonstrated a right-sided pleural effusion associated with mediastinal widening (Figure 1).

**Figure 1:**
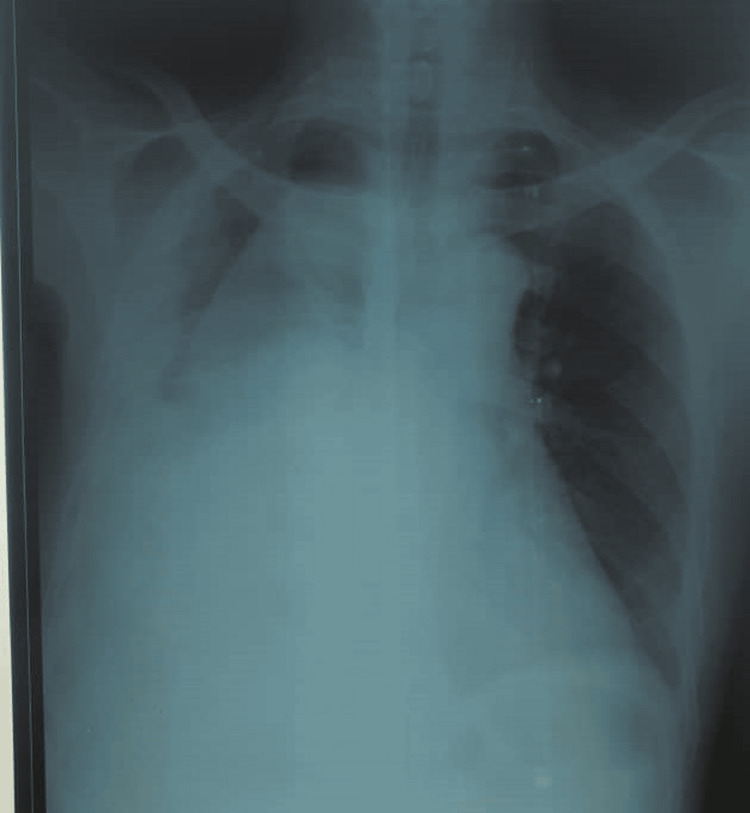
Chest X-ray on admission Chest radiograph demonstrating a large right-sided pleural effusion associated with mediastinal widening, consistent with significant pleural fluid accumulation.

Chest computed tomography (CT) at admission confirmed a right-sided loculated pleural effusion (Figure 2), with pleural thickening and internal septations, consistent with a complex pleural collection initially suggestive of a complicated parapneumonic effusion or empyema.

**Figure 2:**
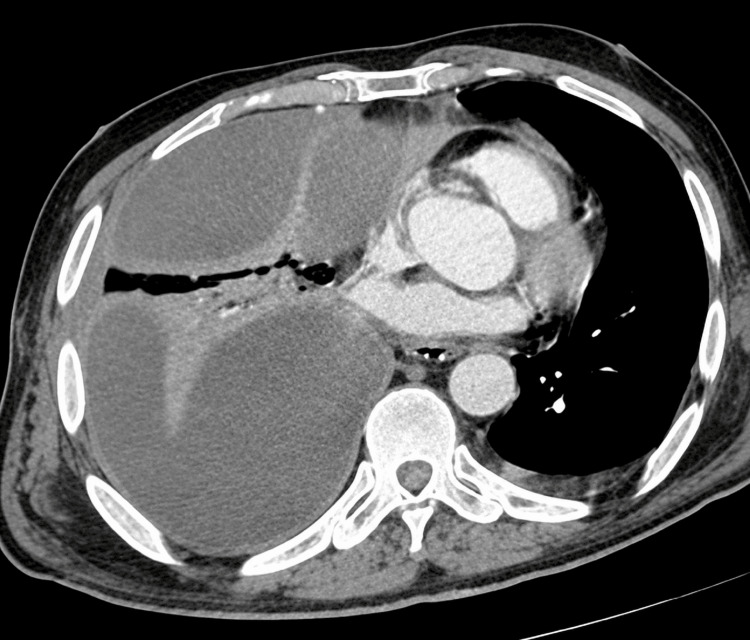
Chest computed tomography at admission demonstrating a right-sided loculated pleural effusion Chest CT at admission showing a right-sided loculated pleural effusion with pleural thickening and internal septations, consistent with a complex pleural collection initially suggestive of a complicated parapneumonic effusion or empyema.

Diagnostic and therapeutic thoracentesis yielded 1400 mL of turbid fluid. Pleural fluid analysis demonstrated an exudative effusion with marked eosinophilia (eosinophils: XX%), elevated protein levels, and increased lactate dehydrogenase, consistent with eosinophilic exudative pleural effusion. Glucose and pH were within non-specific ranges, and adenosine deaminase levels were not suggestive of tuberculosis.

Microbiological investigations, including bacterial cultures, acid-fast bacilli staining, and GeneXpert testing, were negative. Although these findings reduced the likelihood of tuberculosis, pleural TB could not be completely excluded in the absence of pleural biopsy or culture confirmation.

Cytological examination did not reveal malignant cells; however, given the single sampling and the known limited sensitivity of pleural cytology, malignant disease could not be definitively excluded at this stage.

Despite prolonged pleural drainage and empirical broad-spectrum antibiotic therapy with amoxicillin-clavulanate followed by imipenem/cilastatin, no sustained clinical improvement was observed, making complicated bacterial pleural infection less likely. Persistent pleural effusion with fibrinous debris was noted in the drainage fluid (Figure 3), suggestive of a complex inflammatory process.

**Figure 3:**
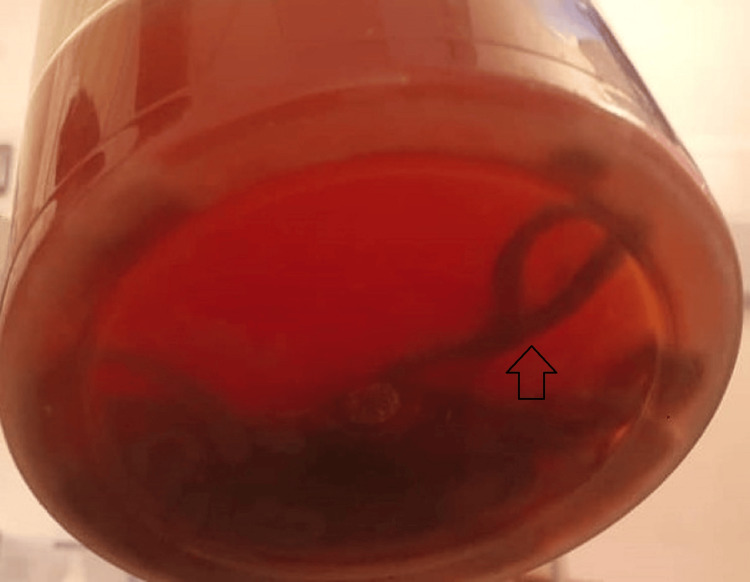
Macroscopic appearance of pleural fluid showing filamentous fibrinous debris Macroscopic examination of pleural drainage fluid revealing filamentous fibrinous material within an exudative pleural effusion, suggestive of a complex inflammatory pleural process.

Given the progressive increase in peripheral and pleural eosinophilia, a parasitic etiology was suspected. Serological testing revealed elevated anti-Toxocara canis IgG levels in both serum and pleural fluid, confirmed by Western blot analysis, establishing the diagnosis of pleural toxocariasis.

Treatment with albendazole led to rapid clinical improvement. Pleural drainage resolved within one week, with progressive radiological regression of the pleural effusion and normalization of eosinophil counts. Follow-up imaging at one and three months demonstrated marked improvement, with significant regression of the pleural abnormalities (Figure 4).

**Figure 4:**
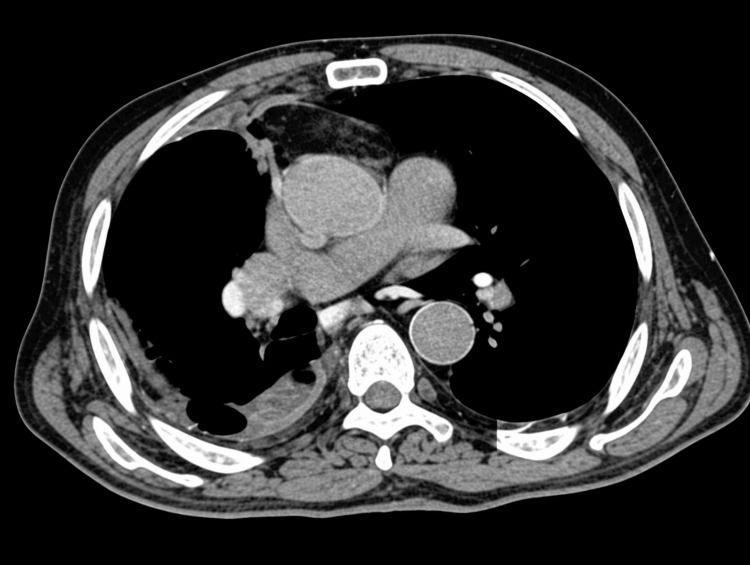
Follow-up chest CT Follow-up chest computed tomography at one month demonstrating marked regression of the right-sided pleural effusion after treatment.

## Discussion

EPE is defined by the presence of ≥10% eosinophils in pleural fluid and represents a relatively uncommon subtype of exudative pleural effusion, accounting for approximately 5-16% of cases [3]. Its etiological spectrum is broad and includes malignancy, infections, pulmonary embolism, drug-induced reactions, and parasitic diseases [3]. Recent studies have challenged the traditional perception of EPE as predominantly benign, demonstrating that malignancy remains one of the leading causes in several series, accounting for a substantial proportion of cases [3]. Therefore, pleural eosinophilia should not be interpreted as reassuring and requires systematic evaluation for both benign and malignant causes.

Parasitic infections are an important but often under-recognized cause of EPE, particularly in endemic and rural settings [3,6]. Several parasites have been implicated, including Paragonimus, Echinococcus, and filarial species, as well as rarely reported etiologies such as toxocariasis [6]. Pleural involvement in parasitic disease remains exceptionally uncommon, with only isolated cases described in the literature [10,11]. These infections may induce pleural inflammation through hypersensitivity reactions or migration of larvae to pleural structures.

Toxocariasis is a worldwide zoonotic infection caused by Toxocara canis or Toxocara cati. Humans acquire infection through ingestion of embryonated eggs from contaminated soil or contact with infected dogs or cats. Pulmonary manifestations are relatively frequent in visceral larva migrans and are typically associated with peripheral eosinophilia, whereas pleural involvement remains exceptionally rare [6,12]. Fewer than 10 cases of pleural toxocariasis have been reported in the literature, highlighting the unusual nature of this presentation [12].

The diagnosis of pleural toxocariasis is challenging due to its nonspecific clinical and radiological features. Patients often present with symptoms suggestive of infectious pleurisy, including fever, chest pain, and dyspnea. Imaging findings are also non-specific and may mimic parapneumonic effusion, empyema, malignancy, or pleural tuberculosis, particularly in high-burden regions [4,13]. In this context, microbiological negativity does not exclude these conditions, and diagnostic uncertainty is common.

In our case, EPE associated with peripheral eosinophilia was a key diagnostic clue. However, pleural eosinophilia is not specific and should be interpreted within the broader clinical context. The persistence of pleural effusion despite appropriate antibiotic therapy and negative microbiological investigations prompted further diagnostic evaluation.

Serological detection of anti-Toxocara IgG antibodies, confirmed by Western blot, supported the diagnosis in this clinical context. However, it is important to note that seropositivity may reflect previous exposure, particularly in endemic areas, and does not in itself confirm active disease. The diagnosis therefore relied on a combination of compatible clinical features, eosinophilic inflammation, exclusion of more common causes, and therapeutic response.

Although the patient showed marked clinical and radiological improvement following albendazole therapy, this response should be interpreted as supportive rather than definitive evidence of causality, as spontaneous resolution or concurrent treatment effects cannot be fully excluded.

Overall, this case highlights several important clinical implications. First, EPE requires systematic and comprehensive etiological investigation. Second, parasitic infections should be included in the differential diagnosis, particularly in rural or endemic settings. Finally, in tuberculosis-endemic regions, clinicians should remain cautious not to attribute pleural effusion to tuberculosis without adequate microbiological or histological confirmation, especially when clinical evolution is atypical or when eosinophilia is present. Pleural eosinophilia should prompt a broad differential diagnosis rather than an infection-centered approach alone.

## Conclusions

EPE remains a challenging clinical entity due to its wide etiological spectrum. This case highlights pleural toxocariasis as an uncommon but important differential diagnosis, particularly in rural and endemic areas. Persistent pleural eosinophilia in the absence of response to empirical antibiotic therapy should prompt consideration of parasitic infections among other causes.

Early recognition and treatment with albendazole may be associated with clinical improvement and resolution of pleural effusion; however, causality cannot be definitively established in a single case, and findings should be interpreted in the appropriate clinical context.
